# Drainage versus no drainage after burr-hole evacuation of chronic subdural hematoma: a systematic review and meta-analysis of 1961 patients

**DOI:** 10.1007/s10143-023-02153-7

**Published:** 2023-09-19

**Authors:** Ahmed Aljabali, Aya Mohammed Sharkawy, Belal Jaradat, Ibrahim Serag, Nada Mostafa Al-dardery, Mariam Abdelhady, Mohamed Abouzid

**Affiliations:** 1 Faculty of Medicine, Jordan University of Science and Technology, Irbid, Jordan; 2 Medical Research Group of Egypt, Negida Academy, Arlington, MA USA; 3 Faculty of Medicine, South Valley University, Qena, Egypt; 4 Faculty of Medicine, The Hashemite University, Zarqa, Jordan; 5 Faculty of Medicine, Mansoura University, Mansoura, Egypt; 6 Faculty of Medicine, Fayoum University, Fayoum, Egypt; 7 Faculty of Medicine, October 6 University, Giza, Egypt; 8 Department of Physical Pharmacy and Pharmacokinetics, Faculty of Pharmacy, Poznan University of Medical Sciences, Rokietnicka 3 St., 60-806 Poznan, Poland; 9 Doctoral School, Poznan University of Medical Sciences, 60-812 Poznan, Poland

**Keywords:** Drainage, Chronic subdural hematoma, cSDH

## Abstract

Chronic subdural hematoma (cSDH) is a common neurosurgical condition that can cause severe morbidity and mortality. cSDH recurs after surgical evacuation in 5–30% of patients, but drains may help reduce this risk. We aimed to investigate the effect of drainage versus no drainage on the rates of recurrence and mortality, as well as the clinical outcomes of cSDH. Following the PRISMA (Preferred Reporting Items for Systematic Reviews and Meta-Analyses) guidelines, we searched four electronic databases (PubMed, Cochrane Library, Scopus, and Web of Science) to identify eligible studies reported up to June 2022. Using Review Manager software, we reported four primary outcomes as odds ratios (ORs) and confidence intervals (CIs). The meta-analysis included a total of 10 studies with 1961 patients. The use of drainage was found to be significantly more effective than non-drainage in reducing the “mortality rate” (OR = 0.65, 95% CI 0.43 to 0.97; *P* = 0.04), the “recurrence rate” (OR = 0.39, 95% CI 0.28 to 0.55; *P* < 0.00001), and occurrence of “gross focal neurological deficit” (OR = 0.58, 95% CI 0.37 to 0.89; *P* = 0.01). No significant difference was found in the occurrence of a Glasgow Coma Scale score of 15 (OR = 1.21, 95% CI 0.84 to 1.76; *P* = 0.30). The use of drains after burr-hole irrigation reduces the recurrence, mortality, and gross focal neurological deficit rates of chronic subdural hematomas.

## Introduction

Chronic subdural hematoma (cSDH) is a long-standing blood clot on the brain’s surface underneath its outer coating [1]. Patients with brain atrophy, or the shrinking or withering away of brain tissue due to age or disease, are most likely to develop these liquid clots when they are 60 years or older [2]. Minor head trauma can break blood vessels over the brain’s surface as the brain shrinks inside the skull over time, leading to a steady blood buildup over several days to weeks [1]. The most frequent complaint is a headache, and symptoms might include weakness, nausea, vomiting, lethargy, disorientation, memory loss, nausea, and seizures [1]. Diagnosis involves computed tomography and magnetic resonance imaging brain scans. SDHs vary in density and may extend over a large portion of the brain’s surface. The best therapy method is burr-hole trepanation, which involves surgically draining the hematoma. The most efficient method of treating cSDH is burr-hole craniostomy, which involves evacuation through one or two burr holes drilled over the location of the hematoma [3]. A significant issue with cSDH is a recurrence, which refers to developing another cSDH in the same location. Patients may require additional surgical interventions to address these recurrent hematomas. The use of drains may reduce the likelihood of recurrence; however, their utilization is not commonly practiced [1].

In addition, middle meningeal artery (MMA) embolization for cSDH has been documented regularly in recent years, and several technological advancements to enhance clinical results have been published [4]. After MMA embolization, it has been discovered that embolic materials that are farther distant help prevent recurrences [4]. Abdollahifard et al. performed a meta-analysis of 11 studies and 359 patients. They reported a pooled recurrence rate of 5% (95% confidence interval (CI) 3 to 8%), a need for reoperation rate of 5% (95% CI 3 to 9%), and a peri-procedural complication rate of 4% (95% CI 2 to 9%) following MMA embolization with particle embolic agents [5]. Khorasanizadeh et al.’s retrospective study involved 78 patients and concluded that using coils for endovascular treatment of cSDHs can be as effective as the adjunct use of particle embolization [6]. Investigating the number of MMA branches embolized showed that embolization of the anterior and posterior MMA branches may be associated with an increased likelihood of complete resolution (76%) compared to single-branch occlusion (33%, *P* = 0.014) [7].

Research has shown that using a drainage tube significantly reduces recurrence rates compared to treatments without it [8]. A retrospective cohort study of 102 patients with cSDH showed that the risk of mortality and recurrence was 14.5% and 32% for patients undergoing craniotomy compared to only 8.7% and 17.7% of patients receiving burr hole drainage treatment, respectively [8]. Despite the presence of various drainage methods, subdural drainage remains prevalent. A systematic review and meta-analysis of 15 studies involving 4318 patients analyzed different drainage methods affecting postoperative prognosis. They reported insignificant differences between subdural drainage and subperiosteal/subgaleal drainage groups in recurrence rates (odds ratio (OR)= 1.08, 95% CI 0.83 to 1.42), mortality rates (OR = 1.16, 95% CI 0.92 to 1.48), and postoperative infection rates (OR = 1.08, 95% CI 0.60 to 1.95) [9].

Additionally, Peng and Zhu’s meta-analysis sought to determine if using external drains following burr-hole surgery for cSDH lowers the likelihood of the condition returning [10]. When information from new research becomes available, their findings might alter. Even when the data are pooled, the available research contains too few participants or events to provide a valid conclusion. As a result, some of the studies, thought to be of lesser quality, did not fully define the randomization processes.

Therefore, we conduct an updated systematic review and meta-analysis to compare the effects and safety of using external drains following the burr-hole evacuation to treat cSDH in adults. We compared external subdural drains with no drains following a burr-hole evacuation in randomized controlled trials (RCTs) to manage cSDH in adults.

## Methods

We followed PRISMA statement guidelines when reporting this systemic review and meta-analysis [11]. All steps were done in accordance with the Cochrane Handbook of Systematic Review and Meta-analysis of Interventions (version 5.1.0) [12].

### Eligibility criteria

We included studies in our review if they satisfied the following criteria:

1. Population: patients with subdural hematoma
2. Intervention: drain
3. Comparator: no drain
4. Outcome:

i) Primary outcomes: mortality and recurrence
ii) Secondary outcomes: hospital stay, neurological deficits, and GCS scale

(5) Study design: we included clinical trials, randomized clinical trials, and observational studies (case–control or cohort studies) that are English and involved at least ≥ 10 human patients with subdural hematoma who had drain or no drain operation

We excluded reviews, case reports, editorial letters, conference abstracts, study protocols, animal and phantom studies, and patients who had other treatments before the mentioned operation.

### Search strategy

We searched the following electronic medical databases: PubMed, Scopus, Web of Science, EPSCO, and Cochrane Library from September 2022 to December 2022 using the following query: (chronic subdural hematoma OR cSDH OR subdural hematoma OR subdural hemorrhage OR subdural bleeding) and (drainage OR drain OR drains).

**Fig. 1:**
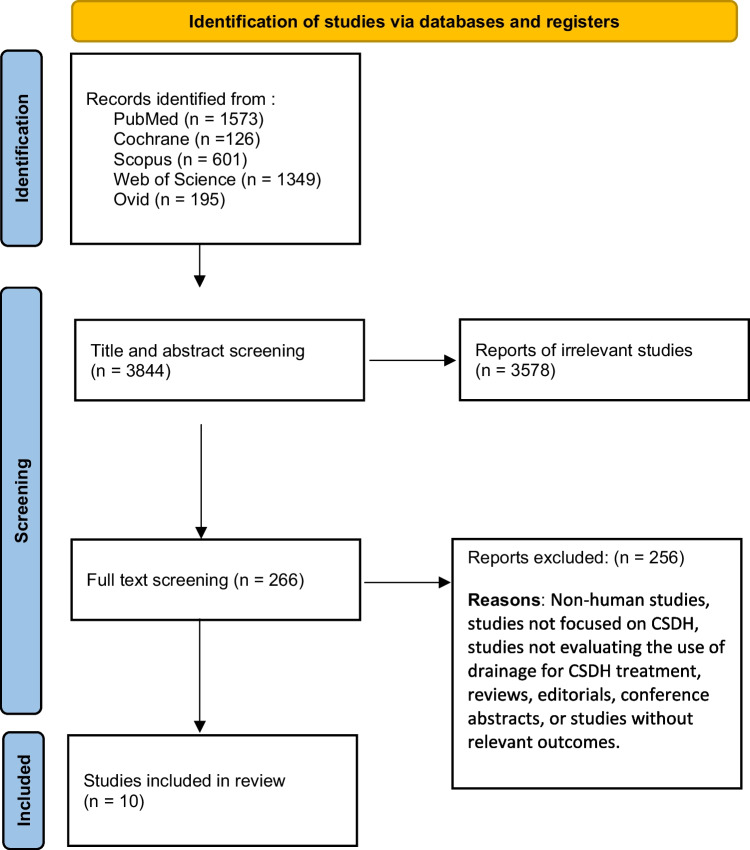
PRISMA chart showing the research strategy and inclusion and exclusion criteria

**Fig. 2:**
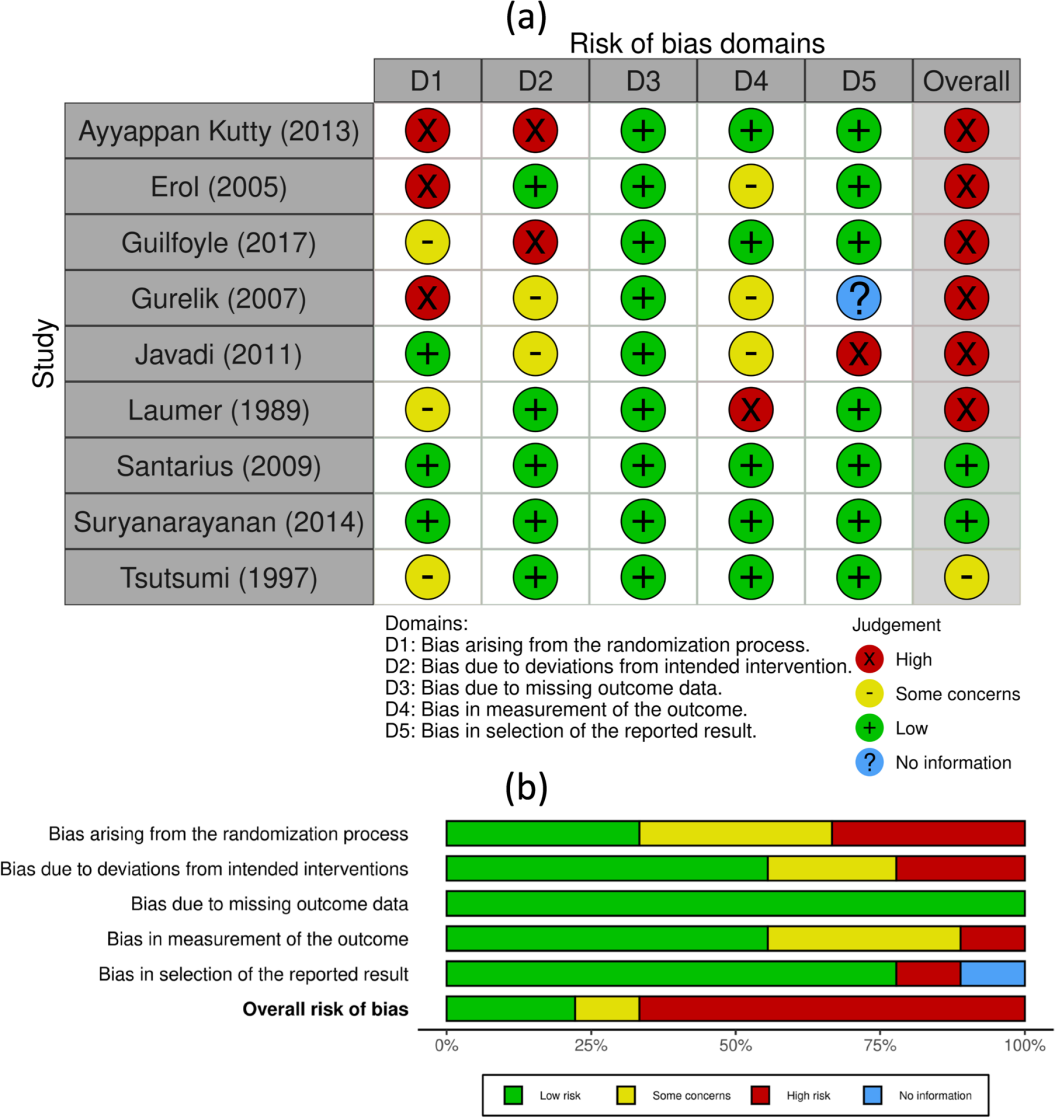
Risk of bias assessment is represented in **a** a traffic light plot and **b** a summary plot according to the Cochrane risk-of-bias tool, created using *robvis* [31]

After retrieving citations from electronic databases, we used Endnote to remove duplicates. Then, the retrieved studies were screened in two steps; the first step was to screen titles and abstracts (on the Rayyan database) of all included references independently by two authors at least to assess their relevance to our meta-analysis, then the next step was to screen the full-text of the identities articles for final eligibility to this meta-analysis (Fig. 1). Then, we extracted data from studies accordingly in a uniform sheet for primary and secondary outcomes and for the risk of bias domains (Fig. 2).

### Data extraction

Three authors extracted data from each paper without dependence on another person or thing and collected them in extraction tables. The following data were extracted from each included study: baseline characteristic of the study population (authors, year of publication, mean age, gender, and total number of patients) and summary of the study included (inclusion criteria and exclusion criteria of each study, study arms and main numbers of patients in each arm, age in each arm, and main findings in each study).

### Quality assessment

Three authors independently assessed the quality of included studies. We use the risk of bias 2 tool and represent data in an Excel sheet; then, risk of bias assessment is represented in a traffic light plot and summary plot according to the Cochrane risk-of-bias tool, created using robvis (Fig. 2).

### Main outcomes

Mortality and recurrence were represented as primary outcomes to know the effect of drainage versus no drainage on the recurrence and mortality rates. Hospital stay, neurological deficits, and GCS scale are secondary outcomes.

### Data synthesis

All outcomes were dichotomous and were presented as event and total. The event and total were pooled using Review Manager (version 5.4) in the random model. An outcome with a *P* value less than 0.05 was considered a significant difference between the two groups. As a diagnostic test accuracy, the estimated overall effect was calculated by MedCalc statistical software.

### Assessment of certainty and heterogeneity

**Fig. 3:**
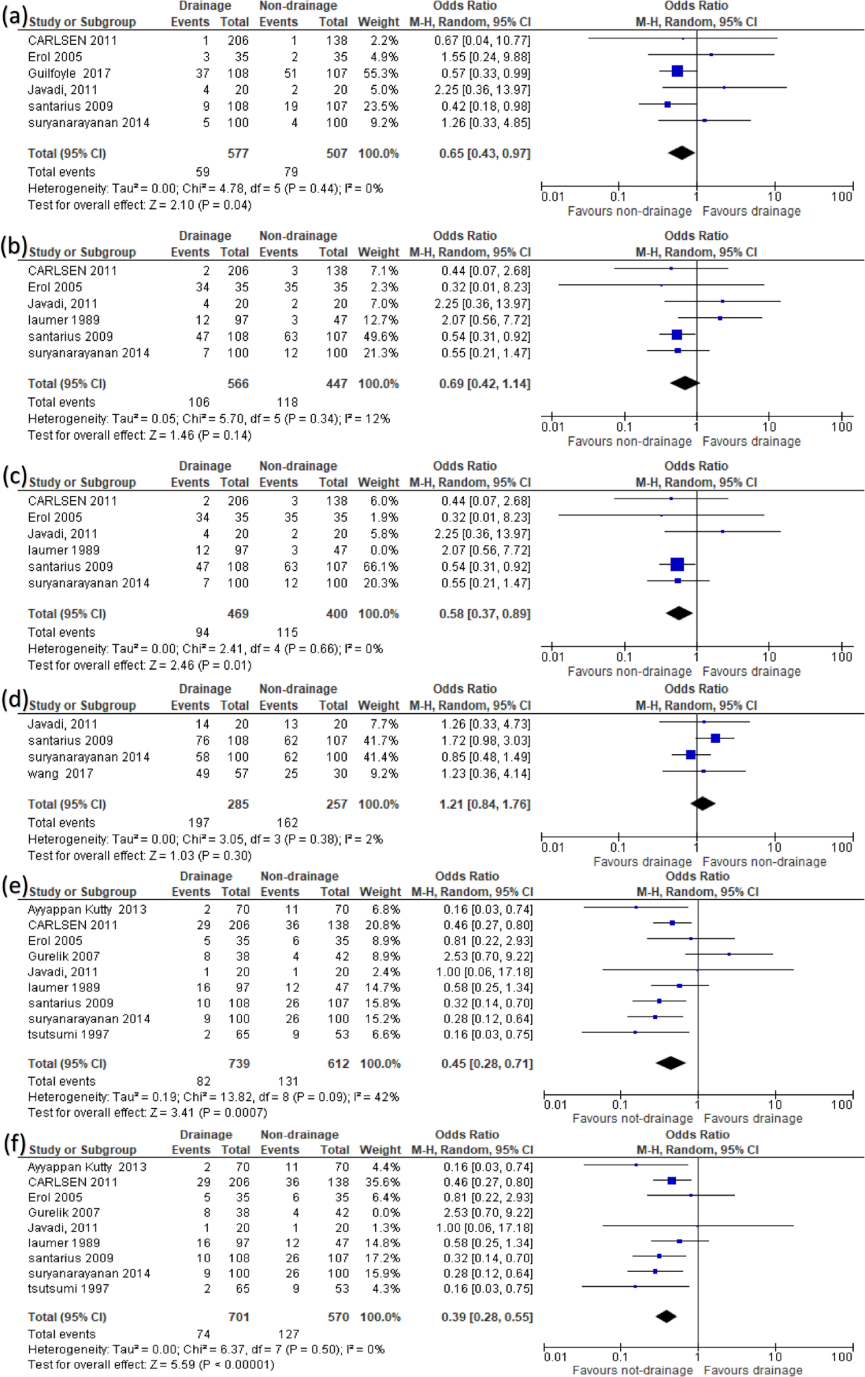
Random-effects models of the risk ratio for primary outcomes: **a** mortality, **b** gross focal neurological deficit, **c** sensitivity analysis of gross focal neurological deficit, **d** GCS of 15 at discharge, **e** recurrence, and **f** sensitivity analysis of recurrence

Sensitivity analysis was used to conduct a certainty assessment. We excluded one study per time to check the strength of the evidence and ensure the overall results were not altered. We included sensitivity analysis for studies showing different results though sensitivity analysis (Fig. 3). Regarding the heterogeneity, the chi-square test evaluated statistical heterogeneity among studies. Then, the chi-square statistic was used to calculate *I*-squared. Chi-square with a *P* value less than 0.1 was considered as significant heterogeneity. Also, the *I*-square value of more than or equal to 50% indicated high heterogeneity.

### Publication bias

**Fig. 4:**
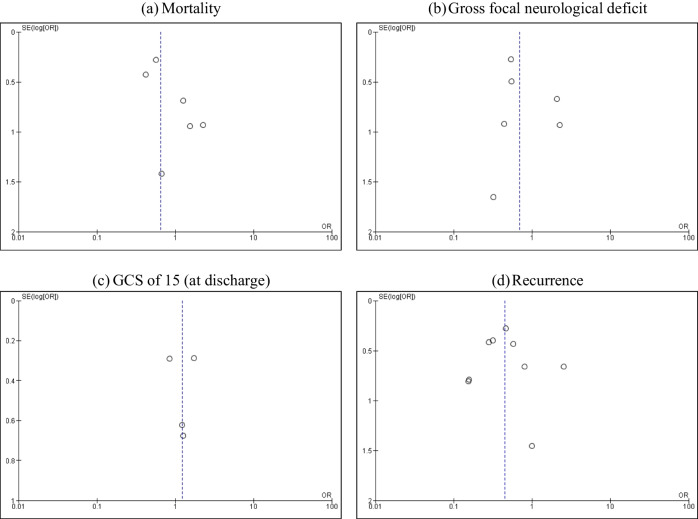
Funnel plots showing the relationship between effect size and standard error for **a** mortality; **b** gross focal neurological deficit; **c** GCS of 15 (at discharge); and **d** recurrence

To explore the publication bias across studies, we constructed funnel plots to present the relationship between effect size and standard error using Revman version 5.4. Symmetrical plots indicated no publication bias, while asymmetrical plots revealed publication bias (Fig. 4).

### Ethical approval

We did not need ethical approval as we collected data from previously ethically approved published studies.

## Results

### Literature search results and study characteristics

Our search yielded 3844 citations. Of these, 266 full-text articles were retrieved and screened for eligibility after excluding irrelevant studies (*n* = 3578). Finally, 10 studies (*n* = 1961 patients) were included in our systematic review [13–22]. Of these, Carlsen et al. [16] was an observational study and eligible to pool in the meta-analysis (*n* = 344 patients), and nine [13–15, 17–22] studies were prospective clinical trial studies (*n* = 1617 patients; the PRISMA flow diagram in Fig. 1). Additionally, we manually searched references of the included studies, and further studies were eligible for inclusion. A summary of general characteristics of the included articles (e.g., study ID, country, study design, total number of patients, inclusion criteria, exclusion criteria, and main findings of each study) and baseline characteristics (e.g., age and gender of included population) are shown in Table 1.

**Table 1:** Summary﻿ of the characteristics of the included studies

Reference	Study	Country	Study design	Total no	Inclusion criteria	Exclusion criteria	Study arms		Main findings	Age (mean ± SD, or range)		Male (%)	
							Arm 1 (drain)	Arm 2 (no drain)		Arm 1 (drain)	Arm 2 (no drain)	Arm 1 (drain)	Arm 2 (no drain)
[13]	Guilfoyle (2017)	England	RCT	215	-	-	108	107	- At 5 years following surgery, the drain group continued to have significantly better survival than the no drain patients (*P* = 0.027) - Survival of patients in the drain group did not differ significantly from that of the general population whereas patients who did not receive a drain had significantly lower survival than expected (*P* = 0.0006)	Both age of 78 years (range 35–95)		74	26
[17]	Ayyappan Kutty (2013)	Turkey	RCT	140	All patients with radiologically proven chronic subdural hematoma, who had undergone burr hole evacuation for the same, between May 2005 and April 2011	1. Age less than 18 years 2. Oral anticoagulant use (warfarin) 3. Patients with bleeding diathesis	70	70	- 11 out of 70 in no drain group had recurrence - Only 2 in no drain group had recurrence	Both mean age 64.86 years and a median of 66 years		79	83
[15]	Suryanarayanan (2014)	India	RCT	200	Patients with cSDH	1. Patients with ipsilateral hematomas who had undergone CSF diversion within 6 months of presentation 2. Patients in whom surgery other than burr-hole evacuation was indicated 3. Patients not needing surgical treatment because of size of cSDH or clinical status of patients 4. Patients in whom brain completely surfaced after burr-hole drainage of CSDH	100	100	- The recurrence between the two groups was statistically significant (*P* = 0.002) decrease in draining group - The difference for mortality in the two groups was statistically not significant (*P* = 0.744)	-		-	-
[14]	Santarius (2009)	United Kingdom	RCT	215	Patients with cSDH	1. Patients with ipsilateral hematoma who had been treated within 6 months of presentation with a shunt for cerebrospinal fluid diversion in situ 2. Those in whom surgery other than burr-hole evacuation was indicated 3.Those in whom the operating surgeon judged drain insertion unsafe were also excluded	108	107	- The rate of recurrence was significantly lower in the drain than in the no-drain group - Time-to-recurrence was longer in the drain - Mortality did not differ between groups - A discharge GCS of 15 was recorded in more participants with a drain than in those without	-		77	72
[21]	Laumer (1989)	Germany	RCT	144	Patients with cSDH	-	97	47	- The rate of recurrence was significantly lower in the drain than in the no-drain group - High incidence of infection after operation in external drainage - No difference in incidence of seizure after operation between two groups	-		-	-
[22]	Tsutsumi (1997)	Japan	RCT	90	cSDH was defined as including: 1. The presence of a typical neomembrane 2. Typical liquified blood within the hematoma cavity 3. If following acute SDH, at least 3 weeks had passed	1. Hygromas 2. Infantile cSDHs, calcified or ossified cSDHs 3. Asymptomatic cSDHs	53	37	- The rate of recurrence was significantly lower in the drain than in the no-drain group - The operative methods were correlated with magnetic resonance findings. In the high-intensity group, 1.1% of cSDHs recurred in patients in whom closed system drainage was used and 11.1% in patients without drainage - In the nonhigh-intensity group, 8.1% of cSDHs recurred in patients in whom closed system drainage was used and 23.1% in patients without drainage	-		-	-
[18]	Erol (2005)	Turkey	RCT	70	-	-	35	35	The most common etiological factor was trauma, complete resolution in the early period was higher in group B (burr-hole craniostomy-closed system drainage) compared to group A (burr-hole craniostomy-irrigation) (60% vs. 40%). Recurrence rates were 17% in group A and 14% in group B	0–20, 1; 40–60, 12; > 60, 22	20–40, 3; 40–60, 13; > 60, 19	77% total men in the study	
[16]	Carlsen (2011)	Denmark	Cohort study	344		Difference protocols were used, patients unavailable for follow-up, re-operated because of acute subdural hematoma and primarily operated by craniotomy	206	138	There were no differences in the complication rates. Postoperative drainage reduces recurrence of chronic subdural hematoma without increasing the complication rate	-		-	
[19]	Gurelik (2007)	Turkey	Randomized trial	80	-	-	42	38	- No significant difference between recurrence rates of the two groups - No correlation between recurrence rate and age, sex, hematoma localization, or etiology	58.4	59.2	67	58
[20]	Javadi (2011)	Iran	RCT	40	-	1. Child (18 years) 2. Midline shift 5 mm 3. Postshunt hematoma 4. Organized hematoma 5. Metastatic hematoma 6. Calcified hematoma	20	20	- Loss of consciousness, and headache were the most common presentations - Recurrence occurred in one patient (5%) in burr-hole irrigation without drainage	68 ± 17	65 ± 19	65	75

Abbreviations: *cSDH *chronic subdural hematoma, *CSF *cerebrospinal fluid, *RCT *randomized controlled trial, *SD *standard deviation

### Risk of bias assessment

Prospective clinical trials assessment by Cochrane tool revealed two studies of overall low risk of bias, one study of some concern, and six studies of high risk of bias. Carlsen et al. [16] was of fair quality risk of bias by Newcastle Ottawa scale (data not shown).

### Outcomes

A) Mortality

The overall effect of the analysis of six studies [13–16, 18, 20] favored drainage over non-drainage in decreasing mortality rate (OR = 0.65, 95% CI 0.43 to 0.97; *P* = 0.04), with no heterogeneity (*I*^2^ = 0%, *P* = 0.44) (Fig. 3a).

B) Gross focal neurological deficit

No significant differences were observed with regard to gross focal neurological deficit between the two groups (OR = 0.69, 95% CI 0.42 to 1.14; *P* = 0.14), with mild heterogeneity (*I*^2^ = 12%, *P* = 0.34) (Fig. 3b). The heterogeneity was resolved after excluding Laumer et al. [21] by sensitivity analysis (*I*^2^ = 0%, *P* = 0.66), and a significant association was found between the non-drainage group and gross focal neurological deficit incidence (OR = 0.58, 95% CI 0.37 to 0.89; *P* = 0.01) [14–16, 18, 20, 21] (Fig. 3c).

C) GCS of 15 (at discharge)

No significant differences were observed with regard to GCS of 15 (at discharge) between the two groups (OR = 1.21, 95% CI 0.84 to 1.76; *P* = 0.30), with no observed heterogeneity (*I*^2^ = 2%, *P* = 0.30) [14, 15, 20, 23] (Fig. 3d).

D) Recurrence

The overall effect of the analysis of nine studies [14–22] favored drainage over non-drainage in decreasing recurrence rate (OR = 0.45, 95% CI 0.28 to 0.71; *P* = 0.0007), with moderate heterogeneity (*I*^2^ = 42%, *P* = 0.09) (Fig. 3e). The heterogeneity was resolved after excluding Gurelik et al. [19] by sensitivity analysis (*I*^2^ = 0%, *P* = 0.50), and results remained significant in favor of drainage (OR = 0.39, 95% CI 0.28 to 0.55; *P* < 0.00001) (Fig. 3f).

### Publication bias assessment

Visual inspections of funnel plots in terms of mortality, gross focal neurological deficit, GCS of 15 at discharge, and recurrence revealed asymmetry. Therefore, there was evidence of potential publication bias (Fig. 4).

## Discussion

This systematic review and meta-analysis revealed a significant difference in the outcomes of patients with chronic subdural hematoma who received drainage compared to those who did not. The results indicate that drainage is associated with a lower risk of mortality, a lower risk of recurrence, and a lower incidence of gross focal neurological deficits in patients with chronic subdural hematoma. However, we did not observe any differences in GCS scores between the two groups.

Knowledge on cSDH has markedly expanded in recent decades. The molecular factors contributing to the pathogenesis of cSDH primarily involve inflammatory and angiogenic pathways [24]. Following an initial injury, a sequence of intricate cellular and molecular responses ensues, resulting in the development of a highly vascularized external neomembrane within the subdural space. This neomembrane is characterized by the accumulation of blood, blood degradation products, and extravasation fluid. Immune cell migration toward the site of injury is driven by chemotactic factors such as fibrinogen degradation products, eotaxin, platelet activation factor, and CXCL-8 (interleukin-8 or IL-8). Elevated levels of cytokines including IL-6, hypoxia-inducible factor, tumor necrosis factor alpha, and cyclooxygenase-2 may stimulate the secretion of vascular endothelial growth factor (VEGF), a critical mediator of angiogenesis. Additionally, proteases like matrix metalloproteinase play a role in releasing angiogenic molecules stored in the provisional extracellular matrix. Intracellular signaling pathways, notably the Smad pathway, are activated by transforming growth factor beta, sensitizing cells to external stimulation via growth factors and cytokines. The neomembrane’s high levels of VEGF, placental growth factor, and angiopoietin-2 promote increased vascular permeability through signaling pathways like MEK/ERK and JAK/STAT. This results in ongoing extravasation of plasma proteins, contributing to hematoma volume expansion. The limited presence of anti-inflammatory cytokines, such as IL-10 and IL-13, and factors conducive to establishing a functional vessel network, like platelet-derived growth factor, may contribute to the chronicity of the condition [24].

Our study results were consistent with previous studies that investigated the same effect in terms of recurrence rate, showing that drainage was associated with 55% [10], 49% [25], and 64% [26] lower recurrence rates. Regarding poor functional outcomes, patients treated with drainage had better functional outcomes, Alcalá-Cerra et al. [25] reported similar results, but Peng and Zhu [10] reported insignificant differences. Our study is the first meta-analysis to demonstrate that drainage was associated with 35% fewer mortality rates, an observation previously observed as insignificant [10, 25, 26]. This finding may be due to the increased number of patients and studies included in our study, which almost doubled. We did not analyze complications between drainage and non-drainage, a factor in which all previous studies [10, 25, 26] did not find differences. However, we reported insignificant results between the two groups in GCS scores of 15 at discharge. Table 2 compares our results to those from previously published meta-analysis studies.

**Table 2:** A comparison of drainage versus no drainage after burr-hole evacuation of chronic subdural hematoma with previous published studies

	Our study	Peng and Zhu [10]	Alcalá-Cerra et al. [25]	Liu et al. [26]
No. of patient	1961	968	628	273
No. of studies	10	9	7	4
Recurrence	Favor drainage			
	OR = 0.39, 95% CI 0.28 to 0.55, *P* < 0.00001, *I*^2^ = 0%	RR = 0.45, 95% CI 0.32 to 0.61, *P* < 0.0001, *I*^2^ = 38%	RR = 0.51, 95% CI 0.36 to 0.75, *P* = 0.0005	OR = 0.36, 95% CI 0.21–0.60, *P* < 0.001, *I*^2^ = 0
	Favor drainage	Insignificant		
Mortality	OR = 0.65, 95% CI 0.43 to 0.97, *P* = 0.04, *I*^2^ = 0%	RR = 0.78, 95% CI 0.45 to 1.33, *P* = 0.35, *I*^2^ = 22%	RR = 0.67, 95% CI 0.37 to 1.22, *P* = 0.19, *I*^2^ = 18%	OR = 0.99, 95% CI 0.45 to 2.16, *P* = 0.98, *I*^2^ = 0
	Favor drainage	Insignificant	Favor drainage	
Poor functional outcomes	OR = 0.58, 95% CI 0.37 to 0.89, *P* = 0.01, *I*^2^ = 0%	RR = 0.68, 95% CI 0.44 to 1.05, *P* = 0.08, *I*^2^ = 31%	RR = 0.61, 95% CI, 0.39 to 0.98, *P* = 0.04, *I*^2^ = 1	-
		Insignificant		
Complication	-	RR = 1.15, 95% CI 0.77 to 1.72, *P* = 0.5, *I*^2^ = 0%	RR = 1.28, 95% CI 0.78 to 2.11, *P* = 0.33, *I*^2^ = 0%	OR = 1.6, 95% CI 0.92 to 2.78, *P* = 0.09, *I*^2^ = 1

It is worth noting that there are many other factors that need to be considered besides the presence or absence of a drain. The optimal orientation for drain placement to minimize pneumocephalus has been investigated in a limited number of studies. For instance, Nakaguchi et al. conducted research and discovered a notable decrease in postoperative pneumocephalus and a reduced recurrence rate in patients in whom the drain was directed anteriorly, specifically toward the frontal region, in comparison to patients with drains placed parietally or occipitally [27]. The rationale behind these findings was attributed to the improved ability to evacuate air collections with a frontal drain orientation, particularly when patients were in a supine position. In another study by Shiomi et al. it was observed that patients with a frontal drain orientation experienced a significantly longer duration before recurrence, and there was a trend toward a lower recurrence rate [28]. Conversely, Ohba et al. conducted their own investigation but did not identify a statistically significant distinction between drain placements in the frontal versus dorsal positions [29]. Additionally, Katsuki et al. noted a correlation between the outer membrane colors (white, red, and yellow) and the histopathological staging from type I to IV, suggesting that the presence of a white outer membrane may pose a risk for recurrence [30]. These findings underscore the significance of comprehending the pathology of cSDH and its connection to endoscopic and surgical observations.

Finally, one of the main strengths of this study is the large sample size, which increases the reliability of the findings. Additionally, the use of meta-analytic techniques allowed us to pool data from multiple studies and synthesize the results statistically rigorously. This helped increase the study’s power and reduce the risk of type I errors. However, some limitations to this study should be noted. First, there is a mild heterogeneity in the included studies, which could limit the generalizability of the findings. This heterogeneity could be due to differences in patient populations, surgical techniques, and postoperative management strategies. Another important consideration is the quality of the studies that were included. Although the inclusion criteria for this study were strict, there was still significant variability in the quality of the included studies. This could have influenced the results and limited the findings’ generalizability.

## Conclusion

This systematic review and meta-analysis prove that drainage is a superior treatment for chronic subdural hematoma compared to no drainage. These findings have significant implications for clinical practice and suggest that surgical drainage may benefit patients with chronic subdural hematoma. However, additional high-quality studies are necessary to validate these results and assess the long-term outcomes of this treatment approach in this patient population.

## Data Availability

All data generated or analyzed during this study are included in this published article.
